# Somatic experiencing® for patients with low back pain and comorbid posttraumatic stress disorder – protocol of a randomized controlled trial

**DOI:** 10.1186/s12906-018-2370-y

**Published:** 2018-11-22

**Authors:** Tonny Elmose Andersen, Hanne Ellegaard, Berit Schiøttz-Christensen, Claus Manniche

**Affiliations:** 1 Department of Psychology, University of Southern Denmark, Campusvej 55, 5230 Odense M, Denmark; 2 Spine Centre of Southern Denmark and University of Southern Denmark, Middelfart, Denmark

**Keywords:** Post-traumatic stress, Somatic experiencing®, Pain, Low back pain, RCT

## Abstract

**Background:**

Research has almost exclusively focused on the neck in order to explain the mechanisms of persistent pain after motor vehicle collisions (MVC). However, studies have shown that low back pain after MVC is as common as neck pain. Also, posttraumatic stress disorder (PTSD) is common after MVCs, and evidence indicate that PTSD may be linked to the development of pain and disability. PTSD has even been proposed as “the missing link” for some in the development of chronic low back pain. Unfortunately, PTSD often goes unattended in low back pain rehabilitation and very few randomized controlled studies exists targeting both conditions. Hence, the aim of the present study is to investigate the potential additional effect of the trauma therapy “Somatic Experiencing®” (SE) in addition to physiotherapy (PT) compared to PT alone for patients with chronic low back pain and comorbid PTSD.

**Methods:**

The study is a two-group randomized controlled clinical trial in which participants (*n* = 140) are recruited consecutively from a large Danish spine center in the Region of Southern Denmark, between January 2016 and December 2017. Patients are randomly allocated to one of the two conditions: SE + PT or PT alone. Measurements of effect are carried out at baseline before randomization, post-intervention, 6 and 12 months post-randomization. The primary outcome is a 20% reduction in disability (Rolland Morris Disability Questionnaire) at 6 months post-randomization. Secondary outcomes are: PTSD symptoms, pain intensity, pain-catastrophizing, fear of movement, anxiety and depression.

**Discussion:**

Comorbid PTSD is currently not targeted in back pain rehabilitation although highly prevalent. If the SE intervention shows to have an additional effect on disability and pain, the study is likely to have a positive impact on the management of chronic low back pain and will have immediate clinical applicability.

**Trial registration:**

Current Controlled Trials Registration August 4, 2017: NCT03244046. Retrospectively registered.

## Background

For decades, research has almost exclusively focused on the neck in order to explain the mechanisms of persistent pain after motor vehicle collisions [1]. Indeed, neck pain is common after motor vehicle collisions (MVC). However, large-scale cohort studies have shown that low back pain (LBP) is as common as neck pain with a prevalence of 37% six weeks after a MVC [2, 3]. Also, wide spread pain across body regions are found to account for almost twice as much of pain related disability (60% vs. 34%) as neck pain alone [2]. Moreover, low back pain is also common after sexual assaults [4, 5]. Finally, collision-related characteristics have consistently shown to be poor predictors of pain and disability after minor MVCs [6–8]. Together, these findings indicate that neurobiological changes related to the psychological stress response to the traumatic event may be important mechanisms in the understanding of low back pain. Indeed, psychological distress such as PTSD is common after MVCs and studies find that comorbid PTSD in chronic pain is associated with a more severe symptom profile with respect to disability, pain and psychological distress [6, 9–11]. PTSD has even been proposed as “the missing link” for some individuals in the development of chronic low back pain [10].

The mutual maintenance model [12] and the shared vulnerability model [13] outlines how elevated levels of arousal, attention bias, anxiety sensitivity, catastrophic thinking and avoidance behaviors are mechanisms maintaining both PTSD and pain. Moreover, examining effect modifiers of exercise rehabilitation programs for chronic whiplash, it is indicated that patients with comorbid PTSD respond less well compared to patients without PTSD. Together, these results indicate that a combined treatment of psychotherapy and exercises may be optimal treating comorbid pain and PTSD [14].

Unfortunately, PTSD often goes unattended in low back pain rehabilitation and very few randomized controlled studies exists targeting both conditions. To our knowledge three randomized controlled trials (RCT) exist targeting comorbid PTSD and pain [15–17] and only one has investigated somatic experiencing® (SE) as a method in a mixed trauma population with chronic low back pain [15]. The other two studies are cognitive behavioral therapy (CBT) trials for motor vehicle-related PTSD. Andersen and colleagues [15] found that a brief SE intervention (6–12 sessions) + treatment-as-usual (TAU: 4–12 sessions of supervised exercises for low back pain) significantly reduced PTSD symptoms and fear of movement (kinesiophobia) compared to TAU alone (*n* = 91). However, no additional effect was achieved in relation to disability and pain compared to TAU alone. Beck and colleagues [16] compared group CBT with a minimal contact comparison condition (*n* = 44) and found a moderate reduction in PTSD symptoms for the CBT group. However, change in pain severity was not different from the controls. The second CBT trial was a pilot study (*n* = 26) on whiplash-associated disorders assessing the effect of trauma-focused CBT compared to a waiting list. Opposite, the other studies, a moderate reduction was found in both neck related disability, pain and PTSD compared to the waiting list [17]. However, the results should be interpreted with caution, given the small sample size and no active control condition applied. Also, only change in PTSD symptoms was considered a clinically important effect [17]. In conclusion, it seems that both SE and CBT approached in the context of chronic pain have an effect on PTSD symptoms. However, given the different nature of the previous studies and the lack of a proper control condition, the effect of targeting PTSD symptoms in comorbid low back pain still remains inconclusive with respect to the effect on pain and disability. More studies with sufficient statistical power and a homogeneous trauma population and a proper control condition are needed. In particular, SE needs to be further assessed given that only two RCTs of SE exists [15, 18] and only the study by Andersen and colleagues testing SE in the context of pain rehabilitation.

SE differs from trauma-focused CBT in its focus on interoception and musculoskeletal sensations rather than exposure to traumatic memories and experiences [19–21]. In SE, traumatic memories are targeted indirectly by gradually developing an increasing tolerance for unpleasant sensations and emotions. For these reasons SE resembles techniques applied in mindfulness and acceptance and commitment therapy for chronic pain [22], where training in sustained attention to unpleasant sensations such as pain or difficult emotions are means to stay mindful in the present moment and thereby facilitate new interoceptive experiences that contradict those of overwhelming anxiety and helplessness associated with pain or traumatic experiences [23].

Theoretically, SE may target important mechanisms as described in the mutual maintenance model [12] and the shared vulnerability model [13]. Indeed, the limited evidence indicate that SE is effective for PTSD and perhaps for fear of movement [15] which according to the mutual maintenance model is one of the mechanisms mutually maintaining pain and PTSD. However, results remain inconclusive in relation to pain and pain related disability. The study by Andersen and colleagues [15] has a number of limitations that may explain the lack of an effect on pain and disability. First of all, the brief SE intervention (mean number of sessions = 6) may not have been sufficient. Also, the mixed trauma population with PTSD dating more than 10 years back in time may have limited the effect. Finally, the long follow-up time 12-months post-randomization may also have deflated the results. For these reasons SE still needs to be investigated as a method in a more homogeneous trauma population with comorbid pain and PTSD and with more sessions of SE. Hence, the aim of the current study is to test whether somatic experiencing® (SE) targeting PTSD related to unresolved accident and injury related trauma in combination with supervised exercises for low back pain would reduce pain-related disability.

First, we hypothesize that an additional SE intervention will reduce pain-related disability compared to physiotherapy alone at 6 months follow-up. Secondly, compared to physiotherapy alone, we hypothesize that the additional SE intervention will reduce all secondary outcomes at 6 months follow-up (PTSD, pain, pain-catastrophizing, fear of movement, anxiety, and depression.

## Methods

### Study design and participants

**Fig. 1:**
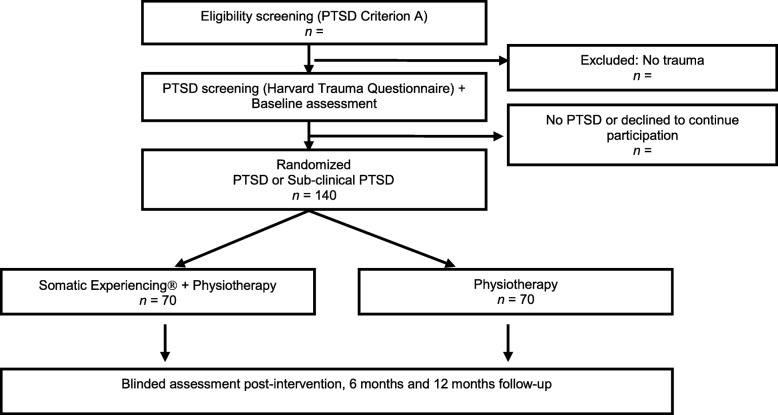
Flow Diagram of the Trial

The study is a two-group randomized controlled clinical trial in which participants (*n* = 140) are recruited consecutively from a large Danish spine center in the Region of Southern Denmark, between January 2016 and December 2017 with follow-up post-intervention, 6 and 12 months post-randomization. Ethics approval was obtained from the local science ethics committee (trial number S-20150136) and all participants gave written informed consent before study entry. Figure 1 illustrates the patient flow in this study. Based on an earlier study in the spine center it is estimated that about 10% will suffer from possible PTSD/sub-clinical PTSD [24]. Of approximately 5000 eligible patients, 500 are expected to have experienced a traumatic event (DSM-IV criteria A [25]) and to screen positive for possible PTSD/sub-clinical PTSD. Of those it is expected that a minimum of 40% will volunteer to participate in the study. In total, 140 patients will be randomized to SE (*n* = 70) and to PT (*n* = 70).

All patient in the spine center are referred for spinal pain and have the right to be referred to the Spine Centre if their improvements has not been satisfactory in primary care. Before study entry, all participants will give written informed consent. Department personnel perform multidisciplinary assessments of patients with spinal pain after referral from general practitioners. A standardized clinical examination and use of magnetic resonance imaging (MRI) are central elements. The clinical examination includes spine movement tests, Lasegue’s test, patellar and Achilles reflexes etc. In addition to the standard screening procedure at the spine center, all patients that have been exposed to traffic accidents or injuries are screened for possible PTSD.

Patients are considered eligible for inclusion if they have low back pain and within 5 years of time has been involved in a MVC or an injury and meet the criteria for possible sub-clinical or clinical PTSD (see procedures below) as measured on the Harvard Trauma Questionnaire part IV [26], are between 18 and 70 years of age, proficient in written and spoken Danish. Exclusion criteria are known psychiatric comorbid diseases such as bipolar depression, psychosis or other serious psychiatric diseases. Also, drug dependence or other ongoing psychotherapeutic interventions will lead to exclusion.

### Randomization and masking

Patients are randomized by random permuted blocks of six. Randomizations are consecutively numbered in sealed opaque envelopes and administered by a project nurse, blinded to treatment assignments. Patients are randomly allocated to one of the two conditions: Somatic Experiencing® (SE) + physiotherapy (PT) or PT alone. Treatment is initiated within 2 weeks after randomization. Measurements of effect are carried out at baseline before randomization, post-intervention, 6 and 12 months post-randomization. The study statistician will be blinded to treatment allocation.

### Procedures

All participants will receive individualized physiotherapy, consisting of supervised functional physiotherapy with the aim to improve daily functioning. The intervention is based on guided exercises for low back pain, psychoeducation in coping with pain and encouragement of daily exercise. No manual treatment approaches will be used. The program will be delivered in 4–8 sessions of 1–2 h. The physiotherapy will be performed by physiotherapists in the center according to the European guidelines for the management of chronic low back pain [27]. Fewer than 4 completed sessions are considered as non-compliance.

### The somatic experiencing® intervention

In the SE intervention, patients receive up to 12 sessions of 1 h SE therapy delivered by a certified SE therapists (a physiotherapist or a pain nurse) with several years of experience in SE and pain management. The SE intervention follows the nine-step model as outlined by Peter Levine [21] and involves gradually eliciting awareness of body sensations associated with the traumatic event. By the process of ‘titration’, patients are gradually encouraged to access, feelings and body sensations as means to restore equilibrium to the autonomic nervous system and thereby alleviate hyperarousal, re-experiencing and avoidance of trauma-related experiences and thoughts. Fewer than 6 sessions are considered as non-compliance.

The overall allowable time frame for the interventions is 16 weeks. The expected timeframe for the final treatment and post-intervention assessment is 12–14 weeks for the SE + physiotherapy group and 8–10 weeks for the group only receiving physiotherapy. An overview of the program is outlined in Table 1.

**Table 1:** Overview of the SE Intervention

Steps	Theme	Therapeutic approach
1	Create a safe environment	To facilitate a therapeutic environment that promotes a feeling of security. Build a therapeutic relationship with the patient. The therapist assumes an accepting stance.
2	Support initial exploration of sensations	To support a mindful approach to the exploration of bodily sensations. Facilitate the experience of positive sensations.
3	Pendulation	By the process of “pendulation” to encourage the patient to come in contact with bodily sensations. To help the client experience how the body alternates between pleasant and unpleasant sensations. By facilitating this awareness, the patient learns how to relax.
4	Restore active defensive responses	To help the patient to restore active defensive responses that has “collapsed” because of the overwhelming nature of the trauma. Support impulses to active responses, including defensive orienting, fight and flight.
5	Titration	Because the central nervous system cannot distinguish between the original trauma and being overwhelmed by the re-experience of the traumatic event in therapy, the aim is to help the patient to gradually move in and out of the trauma. This by ensuring a continuous grounding in the body with attention to the bodily responses while moving into and out of the content of the traumatic event.
6	Uncoupling fear from immobility	Traumatic events activate a flight-fight response. When the traumatic event remains unresolved, the body collapse and becomes “frozen”. The aim is to help the patient to experience this response of immobility in a safe environment and enable immobility to dissolve.
7	Encouraging the discharge of energy	To help the patient to discharge accumulated energy during the traumatic event. Help the client to experience and resolve hyper-arousal states in a safe environment.
8	Restore equilibrium through self-regulation	Through cyclical discharges of energy to help the patient to “reset” the nervous system and feel more empowered to regulate themselves. Allowing time for integration and reverberation.
9	Restoration to the here-and-now	Gently invite the patient to return to the outer world after being attentive to inner sensations and experiences.

*Note.* The nine steps are building blocks and not linear steps. The nine steps are intertwined and may be accessed repeatedly. Adapted from (Levine [21], chapter 5)

### Outcome measures

All outcomes are obtained by an investigator blinded to group allocation at baseline, post-intervention, and at follow-up at 6 and 12 months post-randomization. The outcomes are as follows:

### Primary outcome

The primary outcome is disability 6 months post-randomization measured with the modified version of the Roland Morris Disability Questionnaire (RMDQ-23; [28]). The RMDQ-23 is a self-reported outcome measuring the level of disability related to low back pain. The level of disability is measured on 23 statements covering six different domains: physical ability/activity, sleep/rest, psychosocial level of functioning, household management, eating, and pain frequency. Each statement is scored 1 if the patient feels that the statement is descriptive of their circumstances and scored 0 if not. The total RMDQ score ranges from 0 (no disability) to 23 (maximal disability). Scores are converted to percentages with 23 corresponding to 100% disability. The trial is powered to detect a 20% reduction on the RMDQ from baseline to 6 months follow-up (post randomization). Both internal consistency (Cronbach’s alphaα = 0.84 to 0.96) and test-retest reliability (*r* = 0.83 to 0.91) of the RMDQ are good [29].

### Secondary outcomes

PTSD symptoms are measured using the Harvard Trauma Questionnaire part IV [26]. The Harvard Trauma Questionnaire consists of 17 items with a 4-point Likert scale (1 = not at all to 4 = very often). The 17 items relate to PTSD’s core clusters: avoidance (7 items), re-experiencing (5 items), and hyperarousal (5 items). An item is deemed to be positively endorsed if scores are ≥3. The Harvard Trauma Questionnaire follows the diagnostic criteria for the PTSD diagnosis according to the DSM-IV. In the current study, a possible PTSD diagnosis is proposed if participants report at least one re-experiencing symptom, three avoidance symptoms, and two hyperarousal symptoms. Possible sub-clinical PTSD is proposed in cases where the patients either miss one symptom of avoidance or hyperarousal. The Harvard Trauma Questionnaire self-report measure of PTSD has previously been reported as having an 88% concordance with interview-based estimates of PTSD [26].

Pain intensity is measured as the average score of three 11-point Likert scales measuring peak pain intensity, average pain intensity over the past 2 weeks as well as current pain intensity [30]. Each scale measures pain intensity on a 0–10 numerical rating scale (NRS: [31]) with 0 defined as no pain and 10 as the worst imaginable pain.

The Pain Catastrophizing Scale [32] is used to measure catastrophic thinking related to pain. The PCS ask participants to reflect on past painful experiences, and to indicate the degree to which they experienced each of 13 thoughts or feelings when experiencing pain, on a 5-point Likert scale with (0 = not at all, 4 = all the time). A scale sum score is calculated from all items, with a high score indicating a high level of pain catastrophizing.

Fear of re-injury due to movement is measured with the Tampa Scale for Kinesiophobia (TSK: [33]). TSK is a 17-item scale assessing fear of movement on a 4-point Likert scale ranging from 17 to 68 with higher scores indicating higher levels of kinesiophobia. The scale is commonly used in diverse chronic pain samples and has good construct and predictive validity [34, 35].

To assess the level of depressive symptoms, we used the depression subscale of the Hospital Anxiety and Depression Scale [36]. The HADS was originally constructed to detect anxiety and depression in non-psychiatric medical patients. It was later shown to be useful as a “case finder” also in other populations and it is a well-validated questionnaire with good psychometric properties [36]. The depression scale consists of 7 items related to depression (HADS-D) with responses ranging from 0 (no symptoms) to 3 (maximum impairment).

### Statistics

The sample size of 140 patients was calculated a priori. This sample size provided 80% power to detect a 20% difference in disability on the RMDQ from baseline to 6 months follow-up. This calculation assumed an α of 0.05 and allowed for up to 10% loss to follow up and non-compliance.

The primary and secondary outcomes measured at baseline, post-intervention, 6 and 12 months follow-up (post randomization) will be analyzed using linear mixed-effects models (random coefficient models and multilevel models). With the mixed effects model all data will be used and intention-to-treat analyses applied. Additionally, per-protocol analyses will be applied. Gender and age will be entered as covariates in all models. Assumptions of linearity, homogeneity of variance and normality will be tested.

## Discussion

The primary aim of the present study is to investigate the potential additional effect of a SE intervention in addition to physiotherapy (PT) compared to PT alone for patients with chronic low back pain and comorbid PTSD. The intervention is expected to have an additional positive effect on both disability and all secondary outcomes compared to PT alone. Also, as indicated by earlier preliminary results in whiplash, the comorbid presence of PTSD seems to prevent a good response to physiotherapy [14]. For these reasons, it is believed that an effective treatment of PTSD combined with supervised exercises for low back pain will have an additional effect on both disability and pain. Although Andersen and colleagues [15] only found SE to have a significant effect on PTSD symptoms and fear of movement (kinesiophobia), a number of limitations by the study makes it impossible to draw definite conclusions about the possible additional effect of SE on disability and pain. First of all, the SE intervention was very brief and the trauma symptoms were severely chronic dating back decades. Also, a more structured physiotherapeutic program needs to be tested in combination with a full-length SE program.

The study addresses a prevalent problem, chronic low back pain. Comorbid PTSD is currently not targeted in back pain rehabilitation although highly prevalent after back pain related to injuries. If the SE intervention shows to have an additional effect in disability and pain, the study is likely to have a positive impact on the management of chronic low back pain and will have immediate clinical applicability.

## Abbreviations

CBT: Cognitive Behavioral Therapy
HADS: Hospital Anxiety and Depression Scale
LBP: Low Back Pain
MRI: Magnetic Resonance Imaging
MVC: Motor Vehicle Collision
NRS: Numerical Rating Scale
PCS: Pain Catastrophizing
PT: Physiotherapy
PTSD: Post Traumatic Stress Disorder
RCT: Randomized Controlled Trial
RMDQ: Roland Morris Disability Questionnaire
SE: Somatic Experiencing®
TAU: Treatment As Ususal
TSK: Tampa Scale for Kinesiophobia
